# STAYING ENGAGED: A TAILORED COMMUNITY PROGRAM FOR PEOPLE LIVING WITH DEMENTIA AND THEIR FAMILY CAREGIVERS

**DOI:** 10.1093/geroni/igz038.413

**Published:** 2019-11-08

**Authors:** Gwen McGhan, Kimberly Shapkin, Whitney Alpaugh, Jessica Power Cyr

**Affiliations:** 1 University of Calgary, Calgary, Alberta, Canada; 2 University of Calgary, Calgary, Canada; 3 The Glencoe Club, Calgary, Canada

## Abstract

Tailored care strategies have potential to address declines in physical and cognitive functioning for people living with dementia (PLWD) while making a positive difference in their daily lives; however, these services are not commonplace. As dementia progresses, PLWD become more dependent upon caregivers, increasing caregiving strain and negatively affecting their ability to provide support. The purpose of this study is to examine the feasibility of a tailored community dementia program (TCDP) prescribed for individual abilities of PLWD and measure the impact on the caregiving dyad. A mixed method design was used for a 12-week TCDP with 8 dyads living in the community. Recreational therapy and exercise physiology specialists led the cognitive and physical components of the program. PLWD and caregivers completed assessments at baseline, 6, 12 and 18 weeks. Daily activity was measured in PLWD with no decline observed during the assessment period. Measurements for family caregivers included caregiver strain, satisfaction and assessment of the PLWD’s abilities. Although quantitative findings were not significant, caregivers praised the program in the follow-up focus group with one commenting that “it is important for the mental health of the family and for the patient to keep them active…and (for) socialization”. Another caregiver wrote in their journal “I feel he has really benefited from this program. It keeps him engaged with people and I do think he is more fit”. Overall the TCPD shows promise as a meaningful intervention. Cohort 2 begins in summer 2019 and will implement changes suggested by the caregiving dyads.

